# Global transcriptional analysis of primitive thymocytes reveals accelerated dynamics of T cell specification in fetal stages

**DOI:** 10.1007/s00251-012-0620-6

**Published:** 2012-05-13

**Authors:** Nikolai N. Belyaev, Judit Biró, Dimitrios Athanasakis, Delmiro Fernandez-Reyes, Alexandre J. Potocnik

**Affiliations:** 1 Division of Molecular Immunology, MRC National Institute for Medical Research, London, UK; 2 Division of Parasitology, MRC National Institute for Medical Research, London, UK

**Keywords:** Gene profiling, T cell development, Lineage specification, Hematopoiesis

## Abstract

T cell development constitutes a multistage process allowing the dissection of events resulting in cellular commitment and functional specification in a specialized microenvironment. This process is guided by the appropriate expression of regulatory genetic factors like transcriptional activators or repressors which are, in part, dependent on instructive signals of the microenvironment. To date, it remains unclear whether exactly the same genetic mechanism acts in adult compared to fetal T cell development. In order to directly compare T cell commitment during adult and fetal differentiation, we isolated subsequent stages of intrathymic subpopulations starting with early canonical T cell progenitors up to irreversibly committed T cell precursors. The genome-wide analysis revealed several distinct gene clusters with a specific pattern of gene regulation for each subset. The largest cluster contained genes upregulated after transition through the most primitive pool into the next transitory population with a consistently elevated expression of elements associated with ongoing T cell fate specification, like *Gata3* and *Tcf7*, in fetal progenitors. Furthermore, adult and fetal T cell progenitors occupied distinct “transcriptional territories” revealing a precise land map of the progression to final T cell commitment operating in different developmental windows. The presence and/or elevated expression of elements associated with an ongoing establishment of a T cell signature in the most primitive fetal subset is highly suggestive for an extrathymic initiation of T cell specification and underlines the fundamental differences in fetal versus adult lymphopoiesis.

**Electronic supplementary material:**

The online version of this article (doi:10.1007/s00251-012-0620-6) contains supplementary material, which is available to authorized users.

## Introduction

The hematopoietic system provides an exceptional platform to study cellular differentiation since developmentally arranged populations with prospective functional potential could be identified and isolated. Originating from a hematopoietic stem cell (HSC), which can sustain the life-long production of all blood cells, hematopoietic development proceeds by several distinct maturation steps characterized by stepwise restriction of alternative lineage options and the accompanying acquisition of functional competence. A particular sequence of developmental steps is necessary for the generation of T cells. Functional maturation of T cells is compartmentalized to a specific anatomical site, namely, the thymus, where T cell differentiation takes place in highly specified stromal compartments (Petrie and Zúñiga-Pflücker 2007). T cell precursors—not necessarily restricted to the T cell lineage—seed the thymus at an early stage of their development (Ceredig and Rolink 2002; Shortman and Wu 1996). Further differentiation of T cell progenitors depends crucially on the thymic stroma which is exemplified by the absence of a functional T cell repertoire in “nude” mice carrying a mutation in the forkhead transcription factor *Foxn1* (Nehls et al. 1994). Therefore, intrathymic development presents a paradigmatic model for the mechanism underlying the stepwise restriction of the cellular potential of a progenitor population.

The developmental stages of T cell generation are classified based on expression of two co-receptors, CD4 and CD8 with the earliest intrathymic progenitors expressing neither molecule. These double negative (DN) populations can be further subdivided into four distinct subsets according to their expression of CD44 and CD25 (reviewed in Carpenter and Bosselut 2010; Ceredig and Rolink 2002). The most primitive population, termed DN1, maintains, to some extent, lineage plasticity which is subsequently restricted to achieve full and irreversible commitment to the T cell lineage in DN3. In this subset, the elements of pre-T cell receptor (TCR) as well as Notch-target genes are strongly expressed at the transcriptional level (Taghon et al. 2006; Tydell et al. 2007) marking, together with extensive rearrangements in the TCR β, γ and δ chain loci, the final specification of the T cell lineage at the molecular level. Hence, cell fate is specified precisely during the intrathymic transition from DN1 to DN3. Since the thymus has no capacity for self-renewal of T cell precursors under homeostatic conditions, it relies on the immigration of hematopoietic progenitors originating from the bone marrow in the adult or the fetal liver during ontogeny. The nature of these progenitors that colonize the thymus has been the topic of extensive investigation. Unequivocally, a subset of DN1 expressing the receptor tyrosine kinase c-Kit (CD117) constitutes a canonical T cell progenitor termed “early thymic progenitor” (ETP) (Matsuzaki et al. 1993; Allman et al. 2003). Unfortunately, the exact cellular nature and contribution—both in terms of quality and quantity—of cells recruited to the thymus remain unclear. Several populations present in the adult circulation have been described as potential candidates, including “common lymphoid progenitor”-like cells and various subsets of multipotent hematopoietic precursors (Benz and Bleul 2005; Schwarz and Bhandoola 2004; Umland et al. 2007). During fetal development, a presumably fetal liver-derived progenitor is mobilized, and indeed, some evidence points toward T cell commitment preceding thymus colonization (Rodewald et al. 1994). This potential extrathymic initiation of T cell fate specification in fetal development has been linked to the provision of Notch signals and highlights the pivotal role for this pathway in early T cell differentiation. The importance of Notch signals has been underlined by the observation that induced ablation of the *Notch1* gene resulted in a developmental block at the DN1 stage generating thymic B cells phenotypically resembling immature B cells (Radtke et al. 1999), whereas overexpression of intracellular Notch-1 in hematopoietic progenitors initiates T cell development in the bone marrow at the expense of B cell development, without any obvious defect in myelopoiesis (Pui et al. 1999). However, signals provided by the Notch signaling axis have to act in a well-orchestrated constellation with several other transcriptional regulators. Among those are factors like *Tcf7* (TCF-1) and *Bcl11b*, which are indispensable for early T cell commitment and the subsequent fixation of T cell fate, and at the same time are partly controlled directly by signals provided by Notch (Ikawa et al. 2010; Li et al. 2010a, b; Weber et al. 2011). Other transcription factors known to have critical roles in T cell development like *Gata3* (Hernández-Hoyos et al. 2003), *Gfi1* (Yücel et al. 2003) and *Ikzf1* (Ikaros; (Georgopoulos et al. 1994; Wang et al. 1996)) are additional elements in the establishment of the T cell lineage identity. In summary, alterations in the transcriptional activity of regulatory genes could provide not only one independent criterion to assess the developmental position of a cell during T cell development but also reveal changes in the composition of thymus seeding cells. The direct analysis of this rare cell type in adult and fetal development has been complicated by the potential different nature of these thymus-colonizing cells in the fetus (reviewed in Kincade et al. 2002).

To address the differences between adult and fetal T cell development at the molecular level, we isolated identical stages of early T cell differentiation and profiled their transcriptional activity by microarray analysis. By focusing on purified intrathymic populations, we took advantage of the possibility to align series of “canonical” T cell progenitors in the context of adult and fetal development. Using an unbiased and comprehensive genome-wide approach, we performed a complete transcriptome analysis of the earliest stages of adult and fetal T cell development. This approach enabled us to establish shared or different sets of genes in a model system characterized by the restriction of alternative hematopoietic potential and the establishment of a single cellular identity in a specialized mircoenvironment with minimal interference of irrelevant cell populations. We discuss the usage of this rich informational resource for further identification of molecular players involved in the process of T cell differentiation as a whole but also for recognition of distinct gene clusters that operate exclusively during one developmental window. Furthermore, this information should permit a deeper understanding on the particularities and the nature acting in fetal traits of T cell commitment.

## Materials and methods

### Mice, cell and tissue preparation

Adult female C57BL/6 mice aged 4–6 weeks were kept under sterile pathogen-free conditions at the animal facility of the MRC National Institute for Medical Research, and all animal experiments were done according to institutional guidelines (MRC National Institute for Medical Research Ethical Review Panel) and UK Home Office. For fetal thymi, C57BL/6 mice were mated overnight, and vaginal plaques were recorded the next morning, noon of that day being counted as embryonic day 0.5 (E0.5)

To achieve single cell suspensions of adult and E15.5 thymocytes, thymi were isolated and mechanically disrupted. Cell numbers were determined by counting viable cells using Trypan-blue exclusion in an improved Neubauer chamber.

Lineage depletion was achieved by incubating single cell suspensions with anti-Fc-receptor II/III monoclonal antibody (mab) CD16/CD32 (hybridoma supernatant, clone 2.4G2) to block any non-antigen specific binding of antibodies and subsequent incubation with subsaturating concentrations of biotinylated anti-CD8α (clone 53-6.7), anti-CD3ε (clone 500A2), anti-CD19 (clone 1D3), anti-NK1.1 (clone PK136), anti-CD11b (clone M1/70) and anti-TER119 (clone Ly76) (all eBioscience), followed by a second labeling step with paramagnetic streptavidin conjugated microbeads (Miltenyi Biotech). Microbead labeled cells were finally removed by passing the cell suspension through a magnetic field. The resulting eluent contained ≥ 99 % lineage negative cells as determined by analytical flow cytometry.

### Flow cytometry

Cell concentrations were adjusted to 3 × 10^7^ cells/ml, and staining was performed with allophycocyanin conjugated anti-CD44 mab (clone IM7, eBiosciences), R-phycoerythrin (PE) conjugated anti-CD117 mab (clone Ack45, eBiosciences) and either Pacific Blue conjugated anti-CD25 mab (clone PC61, Biolegend) or alternatively biotinylated anti-CD25 mab (clone PC61, eBioscience), followed by fluorescein isothiocyanate conjugated streptavidin. DN1 ETP, DN2 and DN3 thymocytes were isolated on a MoFlow fluorescence activated cell sorter (Beckman Coulter) or a FACSAriaII (Beckton Dickinson). For RNA preparation, equal numbers of cells were directly sorted into 600 μl TRI Reagent (Molecular Research Center, Inc.). Negative controls were performed using irrelevant isotype-matched control mAbs. Dead cells were excluded from analysis by 7-amino-actinomycin D (7-AAD, Sigma) counterstaining. Analytical flow cytometry was performed on either FACSCantoII (Becton Dickinson) or Cyan (Dako-Beckman) and analyzed using FlowJo software (Tree Star).

### RNA extraction and complementary DNA preparation

RNA was extracted by following the protocol of Chomczynski and Sacchi (1987). Briefly, cells were homogenized in TRI Reagent (Molecular Research Center, Inc.) supplemented with 1/10 v/v polyacryl carrier (Helena Biosciences); phases were separated by supplementation of bromochloropropane (BCP, Molecular Research Center, Inc.) and brief centrifugation. RNA was precipitated from the aqueous phase with isopropanol and washed twice with ethanol. Finally, RNA was air-dried and solubilized at 1 ng/μl. The quality of RNA was assessed on a BioAnalyzer 2100 (Agilent). cDNA was prepared by reverse transcription of the RNA sample using SuperScript II^®^ reverse transcriptase (Invitrogen) according to the instructions of the manufacturer.

### Microarray analysis and data processing

For adult samples, RNA from four independent sorting experiments was pooled to generate the starting material for each individual microchip (i.e. four biological replicates per one technical replicate). For fetal samples, two sorting experiments represent one microarray experiment (two biological replicates per one technical replicate). The starting RNA amount did not exceed 1 μg; therefore, a two-cycle amplification procedure was used, as recommended by the manufacture (Affymetrix). All procedures were conducted by the Microarray Core Facility at the MRC National Institute for Medical Research, London, Mill Hill. Briefly, total RNA was spiked with Poly-A RNA Spike (Affymetrix) consisting of *lys*, *phe*, *thr* and *dap* genes from *B. subtilis*, to later control for amplification efficiency, and reverse transcribed using SuperScript II and a T7-Oligo(dT) Promoter Primer in the first-strand cDNA synthesis reaction (Two-Cycle cDNA Synthesis Kit, Affymetrix). Following RNase H—DNA polymerase I-mediated second-strand cDNA synthesis, the double-stranded cDNA served as a template for the first cycle of in vitro transcription (IVT) (MEGAscript^®^ T7 Kit, Ambion, Inc.). The resulting unlabeled cRNA was then reverse transcribed in the first-strand cDNA synthesis step of the second cycle using SuperScript II and random primers (Two-Cycle cDNA Synthesis Kit, Affymetrix). Subsequently, the T7-Oligo(dT) Promoter Primer was used to generate double-stranded cDNA template containing T7 promoter sequences. The resulting double-stranded cDNA was amplified and labeled using biotinylated nucleotides in the second IVT reaction (IVT Labeling Kit, Affymetrix), to yield labeled cRNA. All reactions were done in a GeneAmp 9700 PCR System (Applied Biosystems). The biotinylated cRNA was cleaned by the Sample Cleanup Modules (Affymetrix), fragmented by metal-induced hydrolysis and hybridized to the Mouse 430A_2.0 GeneChip (Affymetrix). The control oligonucleotide B2 was added to each chip hybridization reaction according to the guidelines of the manufacturer (Affymetrix). The B2 oligonucleotide serves as a positive hybridization control and is used by the GeneChip Operating Software (GCOS) to place a grid over the image in order to define the probe area. In addition, *bioB*, *bioC*, *bioD* and *Cre* were added at specific concentrations to control for hybridization efficiency. Staining with PE-streptavidin and washing were performed using an automated fluidics workstation (Affymetrix), and the arrays were immediately scanned on an Affymetrix GeneChip Scanner, generating an image of the expression data.

The .CELL files [GEO accession number: GSE24142] were analyzed in Genespring GX software (Agilent). Individual arrays were normalized by the GC-RMA algorithm, and the resulting data were first filtered on expression levels of individual probes. Probes with intensity levels above the 20th percentile were taken for further analysis. Subsequently, genes exhibiting a 1.8-fold change between any two conditions were taken for further analysis, and finally, ANOVA was performed to determine the definitive gene list. The Pearson correlation was used to group genes based on their expression levels, whereas K-means clustering was used to group genes based on their expression patterns. To generate population specific transcriptional markers, Recursive Feature Elimination (RFE) with support vector machines (SVM) was performed (Guyon et al. 2002). Briefly, the SVM classification rule for a new point *x* takes the form of the weighted sum $$ f(x) = \sum\nolimits_i {{a_i}k\left( {{x_i},x} \right)} $$, where $$ k\left( {{x_i},x} \right) $$ is a similarity function between points $$ {x_i} $$ and $$ x, $$ referred to as the kernel function. RFE is an iterative variable elimination method specific to SVMs. Once the initial discrimination rule has been inferred by using the SVM on the full set of genes, RFE measures how sensitive the SVM is to the removal of each gene, by measuring how much the removal of the gene alters the resulting classifier. In this way, the method recursively ranks genes by their contribution to the classification rule and eliminates the one that is least informative. By repeating this process over a number of iterations, RFE arrives at a small set of genes, which are important in producing the classification rule and therefore highly representative of each class of data.

## Results

### Phenotypic and proliferative differences between adult and fetal progenitor thymocytes

**Fig. 1:**
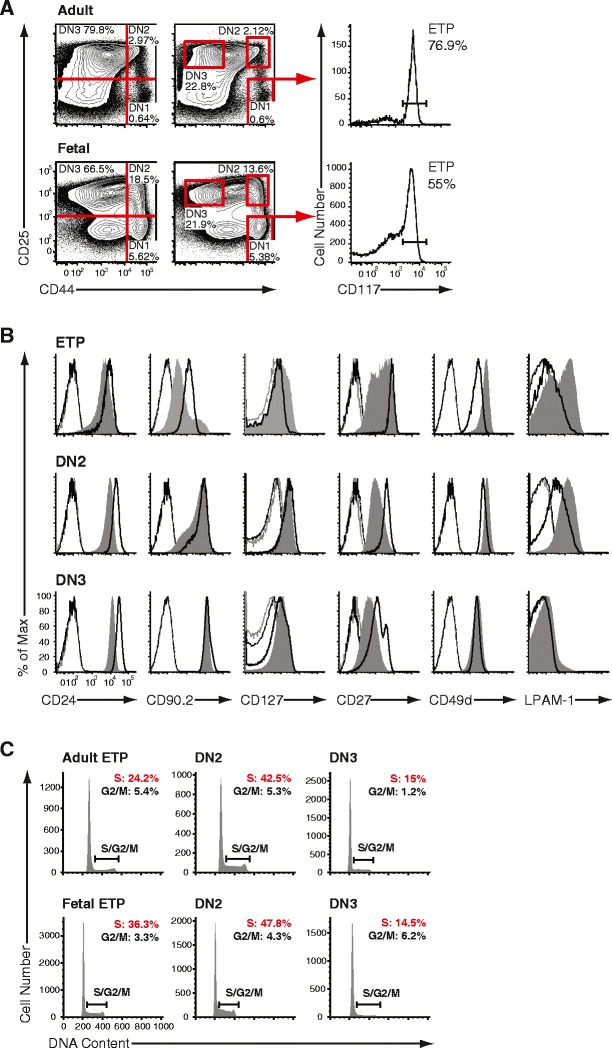
Composition of adult and fetal thymocyte progenitor subsets. **a** Identification of subsequent stages of early intrathymic differentiation by flow cytometry. Representative histograms illustrating cell surface expression of CD44 and CD25 on lineage negative (double negative, DN) thymocytes in adult (*top*) and E15.5 fetal thymus (*bottom*). DN thymocytes were segregated into four populations DN1–4 by expression of CD44 and CD25, as defined by quadrant gating (*left panels*) or by strictly positioned gates to identify more homogeneous subsets (*middle panels*). DN1 was CD44^+^CD25^–^, DN2 was CD44^+^CD25^+^, DN3 was CD44^lo/int^CD25^+^, and DN4 was CD44^lo/int^CD25^–^. Early thymic progenitors (ETPs) were defined as CD117^+^ (receptor-type tyrosine kinase c-Kit) cells in DN1 (*right panels*). The ETP is a major constituent of adult and fetal DN1. **b** Representative histograms illustrating expression of CD24, CD90.2, CD127, CD27, CD49d, LPAM-1 on adult (*black solid*) and E15.5 fetal (*gray filled*) on DN1 ETP, DN2 and DN3 progenitor thymocytes. Isotype matched controls represent negative staining (adult: *faint black*, fetal: *faint gray lines*). **c** Cell cycle analysis of adult versus fetal thymocyte subsets. Representative histograms illustrating the frequency of cells in S/G2/M in adult (*top*) and fetal (*bottom*) DN1 ETP, DN2 and DN3 progenitor thymocytes. DN2 populations contained the highest frequency of cells in cycle, whereas DN3 cells were largely non-dividing

Both adult and fetal (E15.5) intrathymic progenitors were identified according to their expression of CD44 and CD25 in Lin^–^ cells (Fig. 1a). The DN1 compartment was further subdivided into CD117^lo/int^ and CD117^hi^ (ETP) cells, the latter representing all early canonical T cell progenitors (Allman et al. 2003). At first, to establish side-by-side phenotypic differences between adult and fetal T cell development, expression of surface antigens was assessed in ETP, DN2 and DN3 populations (Fig. 1b). T cell differentiation was accompanied by a gradual upregulation of CD24 (heat-stable antigen) and CD90.2 (Thy1.2) from the ETP to the DN3 stage both during adult and fetal developments. CD24 surface levels were slightly elevated on adult progenitors at each step of differentiation as compared to the fetal stages. Expression of CD90.2 was homogeneously intermediate in adult DN1 ETPs, whereas this expression was heterogeneous on the analogous fetal population defining a CD90.2^lo/int^ and a CD90.2^hi^ population. Furthermore, expression of CD127 (interleukin-7 receptor α) showed a noticeable difference between adult and fetal ETPs, with a subset of fetal ETPs having a higher expression of this molecule on their surface. Upon further maturation, expression of CD127 was indistinguishable between adult and fetal subsets, with the DN2 population exhibiting the highest level of surface expression. Distinct expression patterns of CD27, α4 integrin (CD49d) alone or as a complex with β7 integrin (LPAM-1), were evident between adult and fetal progenitors. Interestingly, CD27 did not exhibit a bimodal expression on fetal DN3 as opposed to adult. In addition, fetal cells exhibited generally lower surface expression of CD27 with the exception of ETPs, in which a fraction stained comparable to their adult analog. No evidence resembling the well-regulated pattern of CD27 in adult subsets marked by high surface levels on ETPs, downmodulation in DN2 and part of DN3, and upregulation in successfully selected αβ and γδ T cell progenitors was evident in fetal development. In case of α4 integrin, we observed increased expression of the heterodimers with β1 and β7 integrin on ETPs and DN2 cells. In ETPs, the expression pattern of β7 integrin illustrated further the phenotypical heterogeneity of this subset in fetal development. Given the recent observation that the chemokine receptor CCR9 marks early thymic immigrants (Benz and Bleul 2005) in adult thymopoiesis and is expressed by a large cohort of peri- and intrathymic hematopoietic cells around the onset of thymic colonization at 12.5 days post gestation (Jenkinson et al. 2007), we investigated its expression on adult and fetal ETPs (Supplementary Fig. 1). We found, in agreement with published results (Benz and Bleul 2005), that CCR9 is expressed dimly on a small subset of ETPs in adult and E15.5 fetal thymopoiesis. CCR7 was found to be undetectable both on adult and fetal ETPs. Interestingly, the receptor tyrosine kinase flk-2 (CD135) was present on a subset of adult ETPs, whereas fetal ETPs exhibited just a small shift of the population compared to the relevant control. Taken together, adult ETPs and their fetal counterpart at E15.5 shared a number of common phenotypical features, particularly an already downregulated chemokine expression profile for CCR9 compared to the first wave of thymus-colonizing cells at E12.5.

Next, to further identify developmental differences between sequential stages of adult and fetal T cell developments, we determined the proliferative state of each respective population by measuring the relative DNA content in fluorescence-activated cell sorter (FACS) purified progenitor subsets (Fig. 1c). In adult progenitors, the greatest proliferative capacity was observed among the DN2 population (S/G2/M: 48.5 ± 2.4 %), whereas the DN3 cells exhibited the lowest proliferative capacity (S/G2/M: 15.1 ± 0.9 %). The adult DN1 ETP population contained 22.9 ± 4.3 % of cells in S/G2/M phases. In the fetal progenitors, the dynamics of proliferation exhibited an identical trend, in that the DN2 population showed the greatest proliferative capacity (S/G2/M: 50.2 ± 5.9 %), whereas the DN3 cells were least proliferative (S/G2/M: 20.2 ± 0.7 %). The fetal DN1 ETP progenitor population had 40.5 ± 2.2 % of cells in S/G2/M stages, therefore harboring higher proliferative capacity when compared to adult ETP. In contrast, both fetal and adult DN2/DN3 populations were virtually identical in their cell cycle characteristics. In summary, adult and fetal developing thymocytes aligned based on the expression of developmentally regulated cell surface antigens displayed a variation of other markers associated with migration and differentiation mainly in the earliest compartment—DN1 ETP. By contrast, once cells progressed into DN3, phenotype and proliferative capacity were highly similar.

### Microarray analysis of adult and fetal progenitor subsets

**Fig. 2:**
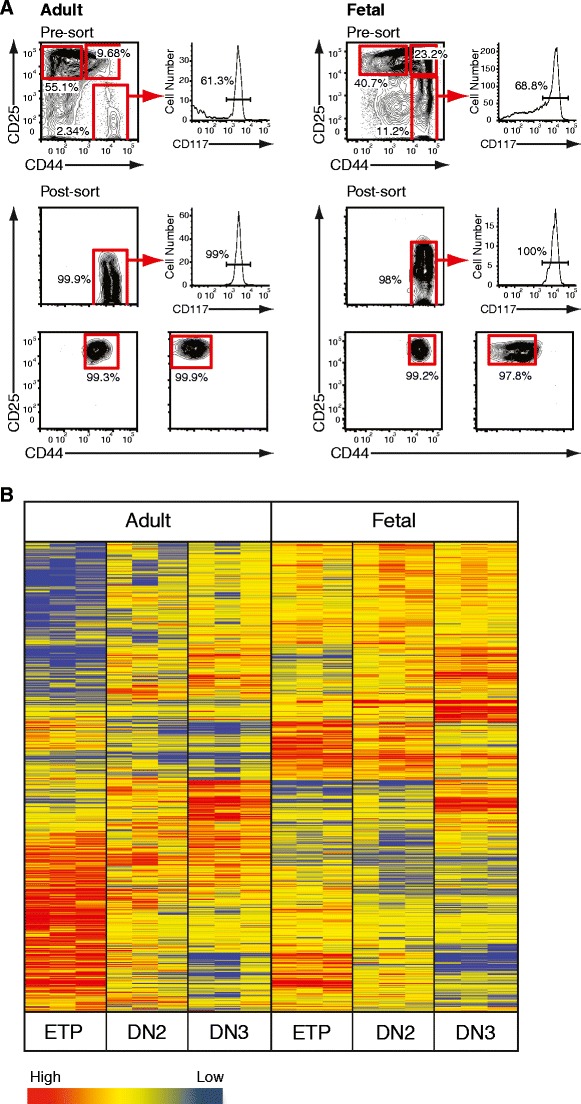
Gene expression profiling of adult and fetal double negative thymocytes. **a** Pre- and post-sort analysis of adult and fetal progenitor thymocytes. Representative histograms illustrating the composition of Lin^–^ DN thymocytes from 4-week-old female C57BL/6 mice and E15.5 embryos as resolved by cell surface expression of CD44, CD25 and CD117 before and after FACS purification. Cells were isolated based on CD44 and CD25 in case of DN2 and DN3 (*bottom panels*). The CD44^+^CD25^–^ DN1 population was further gated on CD117 (c-Kit) positive cells (*middle panels*). **b** Purified ETP, DN2 and DN3 thymocytes from 4–6-week-old female C57BL/6 mice or E15.5 embryos were transcriptionally profiled on the Affymetrix Mouse 430A_2.0 GeneChip. Raw data was transformed and analyzed by Agilent GeneSpring GX 11.0 microarray analysis software. Pearson correlation algorithm generated a heat map illustrating clusters of genes with similar expression levels. Each lane represents an individual technical replicate with pooled material from four (adult) or two (fetal) independently FACS-purified thymic subsets. Individual progenitor populations can be identified by a distinct transcriptional signature

We next established a comprehensive pattern of gene expression accompanying T cell lineage commitment. In order to determine molecular similarities and differences of subsequent stages during fetal and adult differentiation, progenitor populations were FACS purified (Fig. 2a) and subjected to a microarray analysis. Extracted RNA was hybridized to the Affymetrix Mouse 430A_2.0 GeneChip. After normalization probes with intensities less than the 20th percentile were discarded, which left 22,279 probes (annotated list of results normalized to the mean per gene in Supplementary Material Table 1). Subsequently, 7,385 probes fulfilled the criteria of at least a 1.8-fold regulation between subsets and were taken further. Out of these, 4,967 probes passed the filter of *p* < 0.05 after performing analysis of variance (ANOVA) and were therefore included in the final analysis. In order to validate microarray data, real-time PCR was carried out on selected genes which are known to be essential for T cell development and exhibited stringent correlation between the two platforms, thus validating microarray results (data not shown). In order to illustrate global differences in the genetic program underlying T cell development, a heat map was generated using the Pearson correlation algorithm to group genes with similar expression levels (Fig. 2b). This analysis revealed a distinct transcriptional signature for every subpopulation with the most distinct variation in DN1 ETP between adult and fetal development.

### Comprehensive clustering of genes in adult and fetal T cell specification

**Fig. 3:**
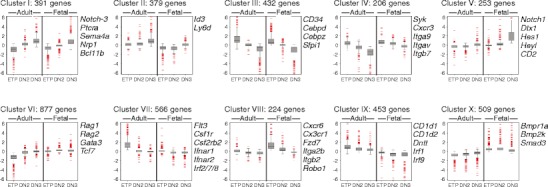
Microarray analysis of adult and fetal double negative thymocytes. K-means clustering revealed ten distinct gene clusters with discrete patterns of expression during adult and fetal early intrathymic development

To permit the unbiased comparison of all differentially regulated genes, we subjected all datasets to K-means clustering and grouped genes with similar expression patterns accordingly (Fig. 3). Firstly, genes that were continuously upregulated between the ETP and the DN3 stages were identified. Two clusters of genes could be separated, which fulfilled this criterion—clusters I and II—only differing in their dynamic range of upregulation (full lists of genes in these clusters are provided as Supplementary Material Table 2). Clusters I and II contained genes with well-established functions in T cell development such as the components of the pre-T cell receptor signaling machinery, namely, *Ptcra*, *Cd3d, Cd3e*, *lck* and *Zap70*; transcription factors *Lef1*, *Ets1* and *Ets2*, *Gfi1*, *Id3*, *Runx1*, *Spib* and notably *Bcl11b*; signal transducers such as *Smo*, *Notch3*; and molecules associated with migration such as *Cdh1* and *Cxcr4*. Genes so far not associated with T cell development found in these clusters were the signal transducer Semaphorin 4A (*Sema4a*) and its ligand Neuropilin-1 (*Nrp1*), representing a potentially novel regulatory pathway. Fittingly, *Cd24*, *Cd90* and *Cd25* were all found in cluster I, perfectly reflecting their pattern at the cell surface. Next, two clusters that exhibited continuous downmodulation of gene expression during progression from ETP to DN3 could be established. Clusters III and IV contained genes that are involved in specification of alternative cell fates (such as *Cebpb*, *Cebpz* and *Sfpi1*—PU.1) ensuring by loss of their transcriptional activity irreversible T cell specification. In addition, clusters III and IV may contain, as yet, unidentified repressors and/or scavenger molecules; downregulation of which would also contribute to the completion of T cell development.

The remaining 67 % of genes were grouped into six distinct clusters all characterized by significant differences between adult and fetal stages in their transcriptional profile. Of particular interest is cluster V which contained genes exhibiting a significantly higher transcriptional activity in fetal DN3 compared to adult DN3. Fixation of T cell identity in fetal development was connected with a strong upregulation of genes implicated in Notch signaling (*Notch1*, *Hes1*, *Dtx1* and *Heyl*). Furthermore, the presence of genes such as *Itga6*, *Itgae*, *Itgb5* and *Cd2* illustrates the difference in the microenvironment that the fetal and adult DN3 cells occupy. Additionally, this cluster is the main candidate for genes connected to emergence of fetal γδ T cells. By far, the largest individual gene set—comprising 20.4 % of all differentially expressed genes—was cluster VI, containing genes that were specifically induced at the transition of adult ETP to DN2, but were expressed in fetal ETPs at significantly higher levels. This cluster contained genes that have been shown to be indispensable for T cell differentiation such as *Gata3*, *Tcf7* and *Rag1/2*. Interestingly, within this cluster, we found the basic-helix-loop-helix transcription factor aryl hydrocarbon receptor gene (*Ahr*). Conversely, we found that cluster VII contained an assembly of genes which were rapidly downregulated upon differentiation from adult ETP to DN2. In fetal thymocytes, a similar regulation was observed, but the expression levels of genes in this cluster were lower as compared with adult. Genes in this cluster are mainly specifically expressed in alternative lineages (*Ica1*, *Wfs1*, *Apbb2*, *Apbb3*) or in early progenitors and/or stem cells (*Gata2*, *Flt3*, *Matk*, *Hoxa5*, *Hoxb4*). The finding that clusters VI and VII together account for a third of all genes (1,443 out of 4,290) further underlined a fundamental difference in the genetic program mainly between adult and fetal DN1 ETPs. Cluster VIII was characterized by a number of genes that might reflect the distinct origin of fetal ETPs and environmental cues that govern migration of fetal progenitors into the fetal thymus. Genes such as *Cxcr6*, *Cx3cr1*, *Itga2b*, *Itgb2*, *Robo1* and *Fzd7* were grouped in this cluster. Finally, clusters IX and X illustrated the presence of global differences in adult and fetal T cell development that would reflect their different lineage outputs, microenvironments and, ultimately, the origin of adult and fetal progenitors.

### Organization of transcriptional territories in thymocyte development

**Figure 4:**
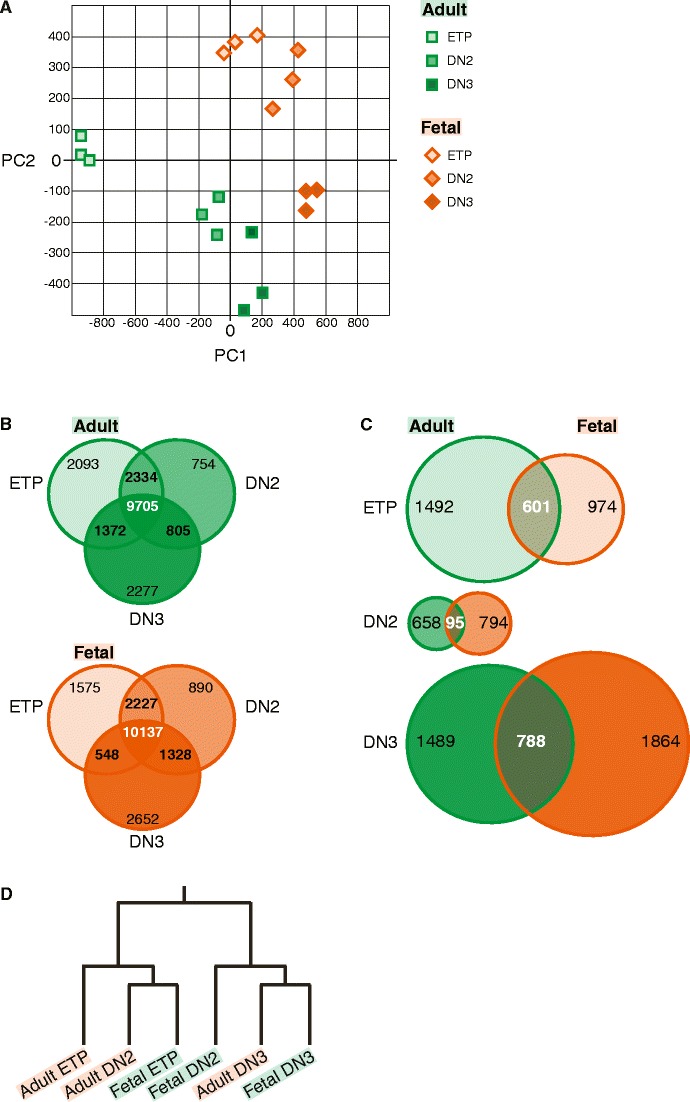
Compartmentalization of adult and fetal progenitor thymocytes according to gene expression signatures. **a** Principal component analysis based on all regulated genes illustrating “transcriptional territories” of adult and fetal T cell ontogeny. Each progenitor population has a distinct molecular signature based on which novel or aberrant hematopoietic progenitors can be classified. **b** Venn diagrams illustrating the overlapping and distinctive genetic elements in adult DN1 ETP, DN2 and DN3 progenitors as well as in analogous fetal subpopulations. Filtered sets of genes were grouped according to their absence or presence of transcriptional activity in every subpopulation. The majority of genes both in adult and fetus were expressed during differentiation; however, each population displayed a unique set of transcribed genes. **c** Venn diagrams illustrating the distribution of genes between adult and fetal DN1 ETP, DN2 and DN3 populations, respectively. Uniquely expressed genes in corresponding populations of adult and fetal development were compared. Analogous populations harbored a set of specific mRNA transcripts that defines each developmental stage. **d** Self-organizing map of adult and fetal progenitor populations. The dendrogram illustrating relationships between adult and fetal progenitors was derived via a self-organizing map. Fetal DN1 ETP and DN2 progenitors exhibited a closer relationship to the final commitment stage (DN3 stage) as compared to the respective adult populations, suggesting earlier specification of the T cell lineage during fetal ontogeny

To further compare the genetic mechanisms between adult and fetal subsets, we visualized all populations based on “two principal components” analysis that best resolved them. Figure 2b clearly illustrates that adult and fetal developmental processes segregated according to their transcriptional programs. Hence, in turn, each specific stage of differentiation could also be separated based on their molecular footprint, thus creating “transcriptional territories” for each developmental stage (Fig. 4a). Remarkably, the establishment of “transcriptional territories” not only separated DN1 ETP, DN2 and DN3 but also resulted in a strict division between fetal and adult populations. This suggests that a distinct collection of genetic elements would be specifically active in an individual stage of development.

In order to address this, we compared the complete transcriptome of each adult and fetal population to identify genes that are specific to the ETP, DN2 and DN3 stages (Fig. 4b). In adult thymopoiesis, 9,705 genes (50.2 % of total) were present in adult DN1 ETP, DN2 and DN3 populations; all of which contained a roughly identical number of genes, whereas a slightly higher number—10,137 genes (52.4 % of total)—was co-expressed during fetal stages of differentiation. There was a large overlap between any two stages of development, but most importantly, each progenitor population had a set of uniquely expressed genes (Fig. 4b). Surprisingly, however, the DN2 population during adult or fetal development contained the least number of unique genes, as compared to the ETP or the DN3 populations, identifying this population as “transitory” during T cell differentiation. To identify sets of genes which were uniquely expressed in any of the six populations, we directly compared transcriptional signatures of corresponding adult and fetal stages (Fig. 4c). This type of analysis revealed that the analogous stages of development in the adult and the fetus did not share majority of unique genes but instead contained a collection of select elements that ultimately define each population. To determine a restricted number of unique genes that reflect the molecular identity of each progenitor population, we performed Recursive Feature Elimination (RFE) with support vector machines and established a distinct set of 8–10 genes that classified each population unequivocally (Supplementary Material Table 3).

Finally, we wanted to illustrate the relationship between the transcriptional programs of adult and fetal progenitors. Transferring the observed expression patterns of regulated genes into a self-organizing map, we found that fetal DN1 ETP exhibited a molecular signature with close resemblance to adult DN2. In consequence, fetal DN2 was grouped closely with finally committed T cell progenitors (adult and fetal DN3, Fig. 4d). Taken together, our results suggest that the genetic program of T cell lineage specification is initiated at an earlier developmental stage during fetal ontogeny resulting in molecular signatures of fetal ETP and DN2 defining more advanced stages of T cell lineage specification relative to adult. In contrast, the closely resembling genetic identity of fetal and adult DN3 cells indicates a largely identical genetic program underlying final T cell fate specification.

## Discussion

In this study, we have used an unbiased approach to investigate the global transcriptional activity during intrathymic T cell fate specification. By focusing on phenotypically defined populations with well-established developmental potential, we compared the genetic processes underlying adult and fetal differentiation and lineage specification in a comprehensive analysis. This approach has generated two main results. First, despite the final restriction of DN3 cells to the T cell lineage illustrated by the identical expression of key components of pre-TCR signaling, a large number of genes were differentially expressed between adult and fetal DN3, illustrating the underlying differences in T cell pools generated during these developmental windows. Second, the mapping of “transcriptional territories” revealed that fetal development is characterized by an accelerated progression towards an endpoint marked by the definitive expression of a T cell specific gene signature in DN3. Furthermore, the fundamental change in the origin and composition of the earliest isolated intrathymic population—DN1 ETP—between adult and fetal was reflected by major differences in gene expression.

The functional capabilities of a living cell are, to a large extent, dictated by the transcriptional programs that operate within. Global transcriptional analyses have allowed for simultaneous detection of thousands of genes, which, in turn, can infer the identity, the origin but, most importantly, the functional capacities of cellular subsets. The hematopoietic system is a prototypic model to study developmentally ordered cellular subpopulations where transcriptional analyses have been employed to gain further insight into the complex regulation of hematopoiesis in wild-type or mutant cells at the global or single cell levels (Akashi et al. 2003; Månsson et al. 2007; Ng et al. 2009). In particular, adult progenitors for the T cell lineage have previously been analyzed to reveal the molecular interplay that accompanies differentiation (Tabrizifard et al. 2004; Tydell et al. 2007; David-Fung et al. 2009; Hoffmann et al. 2003). Moreover, the molecular signals emanating from the adult thymic stromal compartments are also being deciphered by a transcriptomics-based approach (Griffith et al. 2009). We concentrated on the stepwise restriction of cellular potential and the establishment of T cell identity in adult and fetal subsets to reveal a minimal set of genes that are indispensable for T cell differentiation but more importantly to isolate distinct gene clusters that operate in a tight developmental window only. In this study, we employed an improved resolution of the canonical T cell progenitor in DN1 ETP, the correlation of phenotypically aligned adult and fetal samples, and the comprehensiveness of the microarray chip platform. Previous publications using a genome-wide platform for transcriptional profiling either omitted DN1 altogether (Hoffmann et al. 2003) or failed to exclude CD117^–^ cells in DN1 (Tabrizifard et al. 2004), thereby adding subsets to this pool unlikely to contribute to T cell development under physiological conditions. The contamination of the earliest intrathymic progenitor subset with cells not harboring T cell potential in vivo (Tabrizifard et al. 2004) would systematically alter the frequency or abundance of genes not related to T cell differentiation, resulting in a more significant change in the genetic profile upon transition to DN2. Recent studies of E. Rothenberg and coworkers (David-Fung et al. 2006, 2009; Tydell et al. 2007), utilizing an identical isolation protocol for intrathymic progenitors, revealed a pattern of gene regulation similar to the results in our study. Since these reports investigated a large set of regulatory genes based on their initial discovery in cDNA libraries, it is of interest to note that our results in a genome-wide transcriptional screen were in perfect agreement with the reported data. Extending our studies to intrathymic progenitors during fetal development, we succeeded to establish a concise set of genes shared between adult and fetal T cell differentiation. The molecular similarities that were observed should define the prerequisite of genetic activity or, inversely, gene expression silencing, to ultimately generate fully committed T cell progenitors with an irreversible fixation of their developmental potential. Genes upregulated during intrathymic development were found mainly in clusters I, II, V and VI, and they are associated with key events that take place during early stages of T cell development such as Notch signaling (*Notch1* and *3*, *Dtx1*, *Hes1*) and β-selection (*Ptcra*, *Cd3e*, *Cd3d*, *Cd3g*, *Lck*). Of particular interest is the finding that both *Notch1* and *3* were upregulated, but only *Notch1* and its target genes exhibited a different pattern, allowing it to be assigned to cluster V. This could relate to differences in intrathymic Notch signaling between adult and fetal development (Harman et al. 2005) which might reflect changes in the time point and dosage of stimuli. The recent observation that, in adult steady-state thymopoiesis, *Notch1* and *3* have seemingly non-overlapping functions (Shi et al. 2011) further underlines the distinct differences in the regulation of these two Notch family members. Most importantly, the transcription factors *Bcl11b* and *Tcf7* (TCF-1) which are pivotal for the early commitment and specification of the T cell lineage (Ikawa et al. 2010; Li et al. 2010a, b) and, in case of *Tcf7*, which might be induced directly by Notch signaling (Weber et al. 2011) could be found in clusters I and VI. Interestingly, the receptor for interferon γ (IFNγ) and the associated *Stat1* transcription factor were among the upregulated genes found in cluster I among both, adult and progenitor cells, suggesting a potential role for IFNγ signaling in the development or homeostasis of progenitor T cells under steady state. This potential role for IFNγ might relate to the involvement of this particular signaling axis in the generation of a novel bipotent myelo-lymphoid progenitor in acute infection (Belyaev et al. 2010). Another signaling pathway, the neuropilin–semaphorin axis, was revealed among the genes induced (cluster I), implying a potential role of this heterotypic interaction in promoting differentiation of T cells as a whole. In addition, neuropilin has been shown to mark murine regulatory T cells (Sarris et al. 2008); thus, this signaling cascade may be also required in the specification of mature T cell subsets.

Induction of gene expression may serve as an indication of a positive effect on a differentiation process; however, repression of gene activity is equally important mainly regarding the irreversible fixation of a cell lineage identity. One proposed mechanism is the filtration of alternative genetic programs allowing the required pattern of gene expression to dominate, thus yielding the desired outcome (for a detailed review see Rothenberg et al. 2010). This is exemplified in the simultaneous downregulation of genes implicated in the generation of myeloid, B cell and other lineages in adult and fetal T cell progenitors. Coincidentally, downregulation of certain transcription factors that have, as yet, no apparent role in hematopoiesis but function as repressors will also promote T cell development; therefore, clusters III and IV may contain transcription factors being critically involved in the process of developmental commitment to the T cell lineage. Additionally, these two clusters contain common elements that are required for cell adhesion and migration, such as *Cxcr3* and *Itga9*, which could instruct progenitor T cells to gain access to the developmental niche or microenvironment that permits their further development in the thymus. However, the specific requirement in adult and fetal thymopoiesis for migration is best exemplified by clusters VII and VIII, where distinct genetic elements are expressed predominantly in the adult or the fetal DN1 ETP. Among these clusters, chemokine receptors, integrins and a plethora of signal transducers can be found, suggesting different microenvironmental stimuli that progenitor cells integrate and respond to. Of interest in these clusters is the reciprocal expression pattern of *Robo1* and *4* (roundabout homologue receptors-1 and receptors-4) with a clear preponderance of *Robo4* in adult DN1 ETPs, whereas *Robo1* is expressed more preferentially in fetal subsets. Since *Robo4* is expressed in endothelial cells and HSCs and immature progenitors (Shibata et al. 2009), this might hint to their more upstream position in the hematopoietic system as compared with fetal DN1 ETPs. Since members of the roundabout family are crucially involved in the arrest of neuronal migration (Kidd et al. 1998), an attractive speculation on their function during thymocyte differentiation would involve the occupation and/or retention of developing thymocytes in their specific niche. The distinct origin of adult and fetal thymocytes is obvious; adult cells transmigrate from the bone marrow, whereas fetal cells emigrate from the fetal liver. Many studies have provided a wealth of information on the cellular source of adult T cell progenitors (Igarashi et al. 2002; Kondo et al. 1997; Perry et al. 2004), but a fetal counterpart is still poorly defined. Cluster VIII, in particular, but also IX and X, might contain novel markers which could be used to trace the origin and migration of fetal thymic seeding cells. These elements might also—like in case of *Robo1* and *4*—be directly linked with signaling events leading to the induction of migration or retention and homing.

What became apparent from this genetic study is that the transcriptional program associated with the T cell lineage is already initiated in the earliest fetal thymic progenitor as exemplified by cluster VI. The key molecules that are connected to successful T cell specification and development were already expressed in fetal DN1 ETPs, whereas in the analogous adult population, these had to be induced. The organization of adult and fetal progenitors in “transcriptional territories” based on their transcriptional profiles documented that fetal progenitors are grouped more towards the end-stage of T cell lineage commitment—the DN3 stage—as compared to the adult analogues. This implies that the specification and commitment to the T cell lineage is an earlier developmental event in fetal development as compared to the adult, which would have further implications on the nature and mechanism of fetal lymphoid commitment. An alternative explanation would involve the assumption that differences in the transcriptome of early thymocyte subsets are merely the reflection of a varying degree in cellular heterogeneity in adult compared to fetal. In case of DN2 and DN3, we could clearly align the cellular phenotype as well as the proliferative characteristics to a very high degree and yet adult and fetal subsets occupied completely segregated, distinct transcriptional territories. Hence, in these two stages, cellular heterogeneity as defined by the presence of two or more distinctively different cell types is unlikely to explain the differences in the transcriptional program. The degree of cellular heterogeneity is arguably higher in ETPs which contain the thymic seeding cells (TSCs). These thymic seeding cells have been characterized as ETPs expressing flk-2 and CCR9 (Benz and Bleul 2005; Sambandam et al. 2005). As flk-2 expression in fetal E15.5 ETPs did not allow the clear isolation of TSCs, we compared the expression of the chemokine receptor CCR9 in adult and fetal. The ex vivo analysis revealed identical expression profiles on a small CCR9^+^ ETP subset which might represent the cohort of recent thymic immigrants. In view of the equal proportion of adult and fetal CCR9^+^ ETPs, the observed differences in the adult and fetal transcriptome are strongly arguing for either a difference in the extent of T cell commitment with a more advanced status in fetal development or a far higher cellular heterogeneity of adult cells. If heterogeneity could be equated with the possibility that the adult TSC is a complex amalgam of various bone marrow derived populations, we should expect a diverse genetic profile. Interestingly new evidence using a *Il7r*-Cre driven reporter system suggest that the vast majority of ETPs had previously activated the *Il7r* locus, resulting in activating Cre transcription and acquisition of the fluorescent reporter (Schlenner et al. 2010). In conjunction with the observation that the TSC is defined by CCR9 expression, this suggests that the recent thymic immigrant in the adult might, under homeostatic conditions, be less heterogeneous than previously assumed. In light of these recent findings, the isolation of progenitor populations obtained from fate reporter mice in adult and fetal could certainly extend our study by taking advantage of the possibility to further trace lymphoid stages in the bone marrow or fetal liver.

The Notch 1 receptor interaction with its ligands is a crucial event in the specification of the T cell lineage and may be initiated pre-thymically during fetal development (Harman et al. 2005). Another noticeable particularity of this signaling pathway was that *Notch1* along with its target genes were strongly induced in the fetal DN3 population, therefore suggesting a more prominent role of Notch signaling during fetal development and, more interestingly, in signaling through the pre-T cell receptor or the development of the fetal γδ T cells. Lack of CD27 expression, which is thought to mark emerging αβ and γδ T cells in the adult (Taghon et al. 2006), on fetal DN3 cells already points to distinct mechanisms in these processes, and previous identification of progenitor cells that are negative for TCRβ or γδ among fetal DN4 population (Hager-Theodorides et al. 2007) may suggest that the Notch signal alone can be sufficient to drive further differentiation. One important aspect in Notch signaling is its association with leukemogenesis particularly in T cell acute lymphoblastic leukemia. Expression of a number of Notch-induced genes was observed in clinical samples (Palomero et al. 2006; Sharma et al. 2006; Weng et al. 2006; Sanda et al. 2010), and a remarkable similar set of genes was considerably upregulated during fetal differentiation. In conjunction with the more proliferative state mainly of fetal DN1 ETP, this may pre-dispose early fetal thymocytes as prime targets for leukemic transformations.

In conclusion, the unbiased clustering revealed that the significantly elevated expression of lineage-associated transcription factors (*Bcl11b*, *Gata3*, *Tcf7*) and elements of the Notch signaling cascade are indicative for an extrathymic initiation of T cell specification in fetal development. Newly identified populations can be interrogated for the expression of genes relating to each cluster and thus aligned and inserted into the genetic scheme of T cell development. This approach can be extended further to classify already characterized or leukemogenic progenitors and thus construct an ontological scheme of T cell fate specification as a whole based upon transcriptional signatures of populations.

## Electronic supplementary material

Below is the link to the electronic supplementary material.

Table 1 [Suppl_Table_1.xls]: Annotated and normalized list of genes expressed in adult and fetal T cell progenitor subsets. All entries were normalized to the corresponding mean of the gene. (XLS 4853 kb)

Table 2 [Suppl_Table_2.xls]: List of genes clustered according to their expression pattern in adult and fetal T cell progenitor subsets. (XLS 3494 kb)

Table 3 [Suppl_Table_3.xls]: Minimal genetic classification of adult and fetal T cell progenitor subsets. (XLS 20 kb)

Supplementary Fig. 1 Expression of flk-2 and chemokine receptors CCR7 and CCR9 on adult and fetal ETPs. Adult and E15.5 fetal thymocytes were defined according to Figure 1A as Lin^–^ CD25^–^ CD44^hi^ c-Kit^hi^ cells and analyzed for flk-2 (CD135, clone A2F10, conjugated to PE), CCR9 (clone CW-1.2, conjugated to APC and PE) and CCR7 (clone 4B12, conjugated to APC and PE; all eBiosciences) expression by flow cytometry. Results shown are representative for two independent experiments with the tinted blue histogram indicating the isotype-matched control staining. (PDF 81 kb)
