# Supplementary figures and images for: Global transcriptional analysis of primitive thymocytes reveals accelerated dynamics of T cell specification in fetal stages
Source: Immunogenetics. 2012 May 13;64(8):591–604. doi: 10.1007/s00251-012-0620-6 (PMC3395349; doi:10.1007/s00251-012-0620-6)

## ETP adult

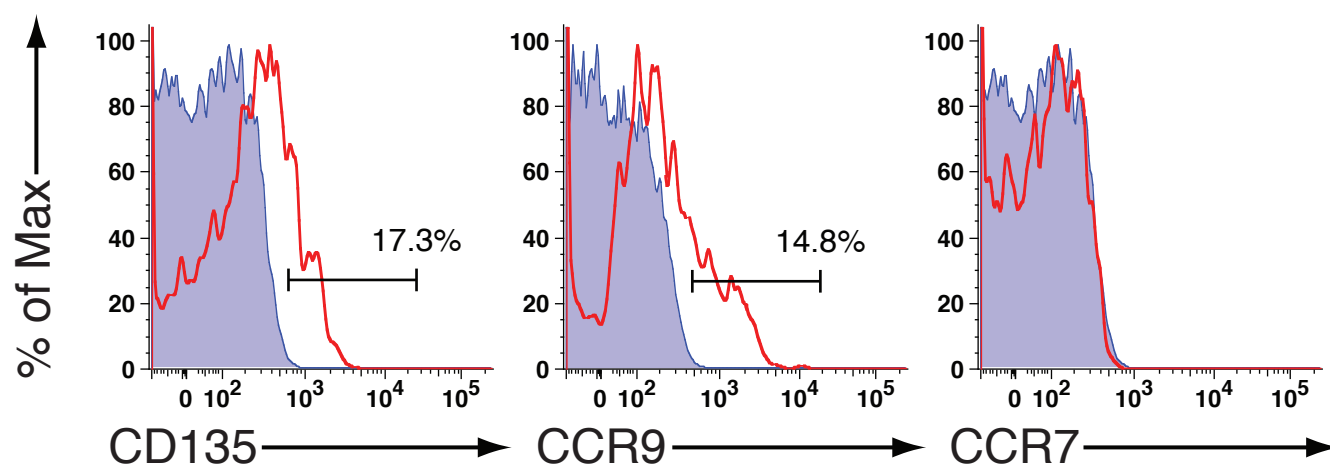

## ETP fetal

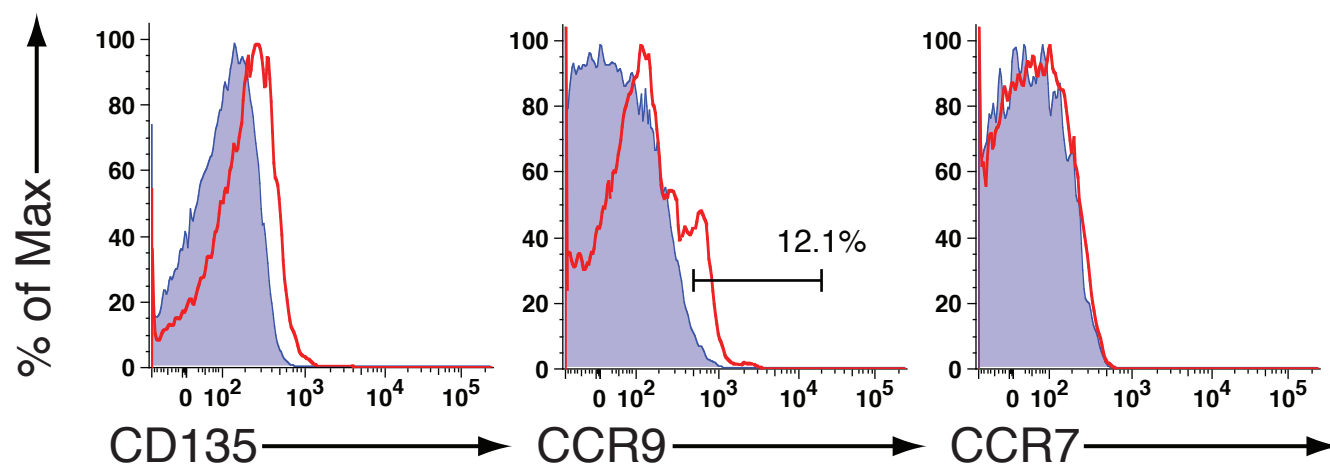

Supplement: Supplementary file 4 — Expression of flk-2 and chemokine receptors CCR7 and CCR9 on adult and fetal ETPs. Adult and E15.5 fetal thymocytes were defined according to Figure 1A as Lin– CD25– CD44hi c-Kithi cells and analyzed for flk-2 (CD135, clone A2F10, conjugated to PE), CCR9 (clone CW-1.2, conjugated to APC and PE) and CCR7 (clone 4B12, conjugated to APC and PE; all eBiosciences) expression by flow cytometry. Results shown are representative for two independent experiments with the tinted blue histogram indicating the isotype-matched control staining. (PDF 81 kb) [file 251_2012_620_MOESM4_ESM.pdf]
